# DHAV 3CD targets IRF7 and RIG-I proteins to block the type I interferon upstream signaling pathway

**DOI:** 10.1186/s13567-023-01134-4

**Published:** 2023-01-26

**Authors:** Xiaoyan Xia, Anchun Cheng, Mingshu Wang, Xumin Ou, Di Sun, Shaqiu Zhang, Sai Mao, Qiao Yang, Bin Tian, Ying Wu, Juan Huang, Qun Gao, Renyong Jia, Shun Chen, Mafeng Liu, Xin-Xin Zhao, Dekang Zhu, Yanling Yu, Ling Zhang

**Affiliations:** 1 Institute of Preventive Veterinary Medicine, Sichuan Agricultural University, Wenjiang, Chengdu, 611130 Sichuan China; 2 Key Laboratory of Animal Disease and Human Health of Sichuan Province, Sichuan Agricultural University, Wenjiang, Chengdu, 611130 Sichuan China; 3 Avian Disease Research Center, College of Veterinary Medicine, Sichuan Agricultural University, Wenjiang, Chengdu, 611130 Sichuan China

**Keywords:** Duck hepatitis a virus, 3CD protein, interferon regulatory factor 7, retinoic acid-inducible gene I, interaction, immune evasion

## Abstract

Duck hepatitis A virus type 1 (DHAV-1) is an acute, highly lethal infectious agent that infects ducklings and causes up to 95% mortality in ducklings up to 1 week of age, posing a significant economic threat to the duck farming industry. Previous studies have found that the proteolytic enzyme 3 C encoded by DHAV-1 can inhibit the IRF7 protein from blocking the upstream signaling pathway of the type I interferon to promote viral replication. However, there are still few studies on the mechanism of DHAV-1 in immune evasion. Here, we demonstrate that the DHAV-1 3CD protein can interact with IRF7 protein and reduce IRF7 protein expression without directly affecting IRF7 protein nuclear translocation. Further studies showed that the 3CD protein could reduce the expression of RIG-I protein without affecting its transcription level. Furthermore, we found that the 3CD protein interacted with the N-terminal structural domain of RIG-I protein, interfered with the interaction between RIG-I and MAVS, and degraded RIG-I protein through the proteasomal degradation pathway, thereby inhibiting its mediated antiviral innate immunity to promote DHAV-1 replication. These data suggest a novel immune evasion mechanism of DHAV-1 mediated by the 3CD protein, and the results of this experiment are expected to improve the understanding of the biological functions of the viral precursor protein and provide scientific data to elucidate the mechanism of DHAV-1 infection and pathogenesis.

## Introduction

Duck hepatitis A virus (DHAV), which belongs to genus *Avihepatovirus* in the *Picornaviridae* family, mainly infects 1-to 4-week-old ducklings, causing neurological symptoms such as corn, spasms, and convulsions. After the autopsy, the ducklings can be shown to have enlarged livers and hemorrhagic spots [1]. Clinically, DHAV can be divided into DHAV-1, DHAV-2 [2], and DHAV-3 [3, 4]. Among them, DHAV-1 is the most prevalent and most toxic. It is popular all over the world and seriously endangers the duck industry. The genome of DHAV-1 is a single-stranded positive-strand RNA containing only one open reading frame (ORF) and encoding only one polyprotein, which is eventually decomposed into structural proteins VP0, VP3, and VP1 by 2 A, 3 C, and 3CD proteases, as well as nonstructural proteins 2A1, 2A2, 2A3, 2B, 2 C, 3 A, 3B, 3 C and 3D [5–7]. The 3CD protein is a unique intermediate in the polyprotein processing cascade, the precursor protein of viral 3 C protease and 3D polymerase.

The innate immune system recognizes virus infection as the first line of defense and induces antiviral responses by producing type I interferons with antiviral, antiproliferative, and immunomodulatory functions. RIG-I-like receptors (RLRs), including retinoic acid-inducible gene I (RIG-I), melanoma differentiation-associated protein (MDA)-5, and laboratory of genetics and physiology gene 2 (LGP2), are a series of cytosolic RNA sensor that detect multiple viral RNAs accumulated during viral infection or replication. Of these, RIG-I senses 5ʹ-triphosphate (ppp) blunt-ended dsRNA [8], while MDA5 is activated by long dsRNA [9]. RIG-I and MDA5 contain two caspase-recruitment domains (CARDs) at their N-terminus, a DExD/H-box helicase domain, and a regulatory domain (RD) at their C-terminus. RNA binding requires intact helicase domains and RDs [10]. After binding of RNA, RIG-I and MDA5 interact with mitochondrial antiviral signaling protein (MAVS) through their CARDs [11] and subsequently recruit inhibitor of nuclear factor kappa-B kinase ε and TANK-binding kinase 1 (TBK1), leading to the phosphorylation and dimerization of interferon (IFN) regulatory factor 3 (IRF3) or interferon regulatory factor 7 (IRF7) [12, 13], and finally, activation of IRF3 or IRF7 enters the nucleus and initiates type I interferon transcription.

Picornaviruses have reported multiple strategies to counteract RLR-mediated antiviral responses. For example, poliovirus (PV) and coxsackievirus B3 (CVB3) 3 C proteins directly cleave RIG-I through their proteolytic enzyme activity [14, 15], and both can also directly cleave MAVS through their 2 A proteins [15]. Foot-and-mouth disease virus (FMDV) 2B protein interacts with RIG-I and induces the reduction of RIG-I to antagonize the RIG-I-mediated antiviral effect [16]. In addition, enterovirus 71 (EV71) inhibits the formation of a complex between RIG-1 and MAVS by interacting with the CARD at the N-terminus of RIG-I, thereby blocking MAVS recruitment and subsequent nuclear translocation of IRF3 [17]. Encephalomyocarditis virus (EMCV) 2 C protein antagonizes the IFN-β signaling pathway by interacting with MDA5 [18].

Although many immune evasion strategies of picornaviruses have been reported, there are still few studies on DHAV-1. Therefore, studying the specific mechanism of DHAV-1 regulating host immunity in antiviral scientific research and clinical application is of great significance. In this experiment, we explored the targets of the DHAV-1 3CD protein regulation of host innate immunity and the mechanisms of interaction, to provide scientific data to elucidate the pathogenic mechanisms of DHAV-1.

## Materials and methods

### Strain and antibody

The DHAV-1 H strain (GenBank accession number: JQ301467.1), the engineered *E. coli* DH5α bacterium, and duck embryo fibroblasts (DEFs) used in this study were provided by the Sichuan Agricultural University Poultry Disease Prevention Research Center. A mouse anti-Flag monoclonal antibody (Cat: M185-3 S) and a mouse anti-HA monoclonal antibody (Cat: M132-3) were purchased from Medical & Biological Laboratories Co., Ltd. A rabbit anti-duck IRF7 polyclonal antibody, a HRP-conjugated goat anti-mouse IgG heavy chain antibody (Cat: AS064), and a HRP-conjugated goat anti-mouse IgG light chain antibody (Cat: AS062) were prepared by ABclonal Technology Co., Ltd. A rabbit anti-beta (β)-actin antibody (Cat: 20536-1-AP) was obtained from Proteintech Co., Ltd. A rabbit anti-HA monoclonal antibody (Cat: AF2305), a mouse IgG antibody (Cat: A7028), and a HRP-conjugated goat anti-mouse IgG (Cat: A0216) were purchased from Beyotime Co., Ltd. A rabbit anti-Histone H3 (Cat: TA6358), a mouse anti-Myc (Cat: T62076M), and a mouse anti-beta (β)-tubulin monoclonal antibody (Cat: T63017-2) were purchased from Abmart Co., Ltd. A rabbit anti-VP3 antibody was prepared in our laboratory [19]. An Alexa Fluor^™^ 568 goat anti-mouse IgG antibody (Cat: A11004) and an Alexa Fluor^™^ 488 goat anti-rabbit IgG antibody (Cat: A11008) were purchased from ThermoFisher Scientific Co., Ltd.

### Plasmids

pCMV-3 C-HA, pCMV, pCAGGS, pCAGGS-3D-HA, pCAGGS-3CD-HA, pCAGGS-RIG-I(N-terminal)-Flag, pCAGGS-TBK1-Flag, IFN-β-Luc, pCAGGS-IRF7-Flag [20], pCAGGS-IFNβ-Flag, and pRL-TK were preserved and provided by the Poultry Disease Research Center of Sichuan Agricultural University. Poly(I:C) was purchased from Sigma–Aldrich. All primers in this study were synthesized by Shanghai Bioengineering Co., Ltd. The primer sequences are shown in Table 1.

**Table 1:** **All primer sequences used in this experiment**

Gene	Primer name	Primer sequence (5ʹ-3ʹ)	Refs.
IFNβ	P1 F	CCTCAACCAGATCCAGCATT	[46] [46]
	P1 R	GGATGAGGCTGTGAGAGGAG	
IRF7	P2 F	CGCCACCCGCCTGAAGAAGT	[20]
	P2 R	CTGCCCGAAGCAGAGGAAGAT	[20]
RIG-I	P3 F	GCGTACCGCTATAACCCACA	[47]
	P3 R	CCTTGCTGGTTTTGAACGC	[47]
β-actin	P4 F	TACGCCAACACGGTGCTG	[46]
	P4 R	GATTCATCATACTCCTGCTTG	[46]
pCAGGS-3*Flag-MAVS	P5 F	GATTACAAGGATGACGACGATAAG CTCGAGATGGGCTTCGCGGAGGACAA	New
	P5 R	TTGGCAGAGGGAAAAAGATCTCTA TTTCTGCAGCCGGGCGTACA	New
pCAGGS-3*Flag-MDA5	P6 F	GATTACAAGGATGACGACGATAAG CTCGAG ATG TCGACGGAGTGCCGAGA	New
	P6 R	TTGGCAGAGGGAAAAAGATCTTCA GTCTTCATCACTTGAAGGACAATG	New
pCAGGS-RIG-I(N-terminal)-Myc	P7 F	CATCATTTTGGCAAAGAATTCGCCACCATGACGGCGGAGGAGAAG	New
	P7 R	TTGGCAGAGGGAAAAAGATCTTCACAGATCCTCTTCAGAGATGAGTTTCTGCTCTCTTATATCCCACAGTTCACT	New

“New” refers to primers designed and synthesized in this paper.

Construction of pCAGGS-3*Flag-MDA5 and pCAGGS-3*Flag-MAVS: upstream and downstream primers were designed to specifically amplify the MDA5 and MAVS fragments (Table 1), and finally, each primer was used to specifically amplify and recover the amplification products. Subsequently, the vector pCAGGS-3*Flag was linearized using the restriction sites for which the primers were designed (Bgl II and Xho I), after which the digested products were recovered, and MonClone^™^ Single Assembly Cloning Mix (Monad) was used for homologous recombination. Positive colonies were identified by PCR using 2×Taq Master Mix (Vazyme), followed by double digestion identification, and the positive clones were sequenced by SangonBiotech (Shanghai) Co., Ltd. The pCAGGS-RIG-I(N-terminal)-Myc construct was performed as described above.

Plasmid preparation and transfection: Endotoxin-free plasmid was extracted from the cloned bacterial liquid containing the target plasmid using Endo-free Plasmid DNA Mini Kit (Omega) according to the instructions, and the plasmid DNA was stored at −20 ℃. Transfections were performed in 6-well culture dishes, 12-well culture dishes, and 24-well culture dishes. The transfection of 6-well and 12-well culture dishes was carried out according to the Hieff Trans^™^ Liposomal Transfection Reagent (YEASEN Biotech Co., Ltd). And the transfection of 24-well culture dishes was carried out according to the instructions of the TransIntro^™^ EL Transfection reagent (TransGen Biotech, Beijing, China).

### Dual-luciferase reporter assay

For this experiment, we referred to the manual of the Dual-Luciferase^®^ Reporter (DLR^™^) Assay System (Promega). Briefly, samples were collected 36 h after transfection, the culture medium was discarded and diluted 1× cell lysate was added, and stored it at −80 °C. 75 µL of the sample was added to a 96-well plate with an all-black transparent bottom, followed by 75 µL of Dual-Glo^®^ fluorophore detection reagent to each well, mixed well and the fluorescence signal was detected to obtain the A value. Added 75 µL of Stop&Glo^®^ detection reagent to each well to detect the fluorescence signal to obtain the B value. Finally, process the data (A value/B value). The resulting data represent the mean and standard deviation of the data from four independent experiments.

### RNA extraction and qPCR

Total RNA was extracted from samples using RNAiso Plus Reagent (TaKaRa) according to the instructions. For cytokine detection, total RNA was reverse transcribed into cDNA using the PrimeScript™ RT reagent Kit (Perfect Real Time) (TaKaRa, Japan) according to the manufacturer’s instructions. SYBR^®^ Premix Ex Taq^™^ II (Tli RNaseH Plus) Kit (Takara) was used to detect the expression of different cytokine mRNA transcript levels in cells. A 10 µL reaction system containing 5 µL SYBR^®^ Premix Ex Taq, 0.5 µL of each upstream and downstream primers, and 1 µL cDNA template was used; the reaction procedure was as follows: pre-denaturation at 95 °C for 30 s; denaturation at 95 °C for 5 s, annealing and extension at 58.6 °C for 30 s for 45 cycles. Viral copy number determination in cells was performed according to the one-step TaqMan fluorescent quantitative RT-PCR method constructed in our laboratory [21, 22]. The relative transcript levels of target genes were analyzed using the 2^− ΔΔCt^ method and compared with the blank control group. All data and images were analyzed and produced using GraphPad Prism 8.0.2 software.

### Western blot analysis

The supernatant was discarded from the transfected cells and the proteins were separated by SDS-PAGE and subsequently transferred to PVDF membranes (Bio-Rad). The membrane was blocked with 5% skimmed milk powder at 37 °C for 3 h and incubated overnight (4 °C) with mouse anti-Flag (1:10 000), mouse anti-HA (1:10 000), mouse anti-Myc (1:1000), rabbit anti-VP3 (1:800), or rabbit anti-IRF7 (1:1000), rabbit anti-β-actin (1:5000), mouse anti-β-tubulin (1:5000), rabbit anti-Histone H3.1 (1:1000) primary antibodies. HRP-labeled goat anti-rabbit IgG (1:3000) or HRP-labeled goat anti-mouse IgG (1:3000) was used as the secondary antibody and incubated with the blot for 1 h at 37 °C. Protein bands were visualized using ECL (Bio-Rad) detection reagent.

### Co-immunoprecipitation experiment

After 36 h of transfection, the cells were lysed with NP40 lysis buffer (Shanghai Biyuntian Biotechnology Co., Ltd.), placed on ice for 30 min and then centrifuged. Take the lysate supernatant and divide it into two parts, and mouse anti-IgG (negative control), mouse anti-Flag, or mouse anti-HA monoclonal antibodies were added at a ratio of 1:100 and incubated at 4 °C for 24 h. Then, added protein A and protein G (Bio-Rad) at a ratio of 1:10 and incubated them at 4 °C for 12 h. Finally, the supernatant was discarded and 40 µL of PBS was added, followed by 10 µL of 5×loading buffer and boiled and subjected to Western Blot analysis.

### Indirect immunofluorescence assay

Cells were transfected according to the appropriate experimental grouping, washed three times with PBS 36 h after transfection, fixed with 4% paraformaldehyde at 37 °C for 3 h, permeabilized with 0.25% Triton-X100 at 4 °C for 25 min, and fixed with 5% BSA at 37 °C for 3 h. The rabbit anti-HA (1:1000) and mouse anti-Flag (1:1000) were used as primary antibodies and incubated overnight at 4 °C, followed by incubation with secondary antibody Alexa Fluor^™^568 goat anti-mouse IgG (1:1000), Alexa Fluor^™^ 488 goat anti-rabbit IgG (1:1000) at room temperature for 1 h. Finally, nuclei were labelled with DAPI (1:1000; D9542, Sigma) at room temperature for 15 min and observed under an inverted fluorescence microscope [23]. Digital images were analyzed as described previously [24, 25], using ImageJ software, to determine the fluorescence intensity above background (Fb) in the nucleus (Fn) compared to that in the cytoplasm (Fc) and to determine the nuclear to cytoplasmic fluorescence ratio (Fn/c).

### Nucleocytoplasmic separation experiment

After 36 h of transfection, cell samples were collected for the nucleocytoplasmic separation experiment. The cell culture medium was discarded and the cells were lysed on ice with NP40 lysis buffer for 30 min. After centrifugation at 12 000 × *g* for 15 min, the cytoplasmic fraction was obtained in the supernatant. The pellets were washed three times with PBS, and 1% SDS lysis buffer was added to lyse the cells on ice for 30 min and collected as cell nuclear fraction. After adding 5× loading buffer, the samples were boiled and detected by Western blotting.

### Treatment of cells with inhibitors of protein degradation pathways

After 24 h of transfection, DMSO and the inhibitors Z-VAD-FMK (50 µM), MG-132 (20 µM), NH_4_Cl (10 mM), and 3-MA (10 mM) were individually added to the cell culture medium. Cell samples were collected after 12 h of treatment with the inhibitors and analyzed by Western blotting.

## Results

### The 3CD protein inhibits poly(I:C)-induced signaling pathway upstream of interferon-β

**Figure 1:**
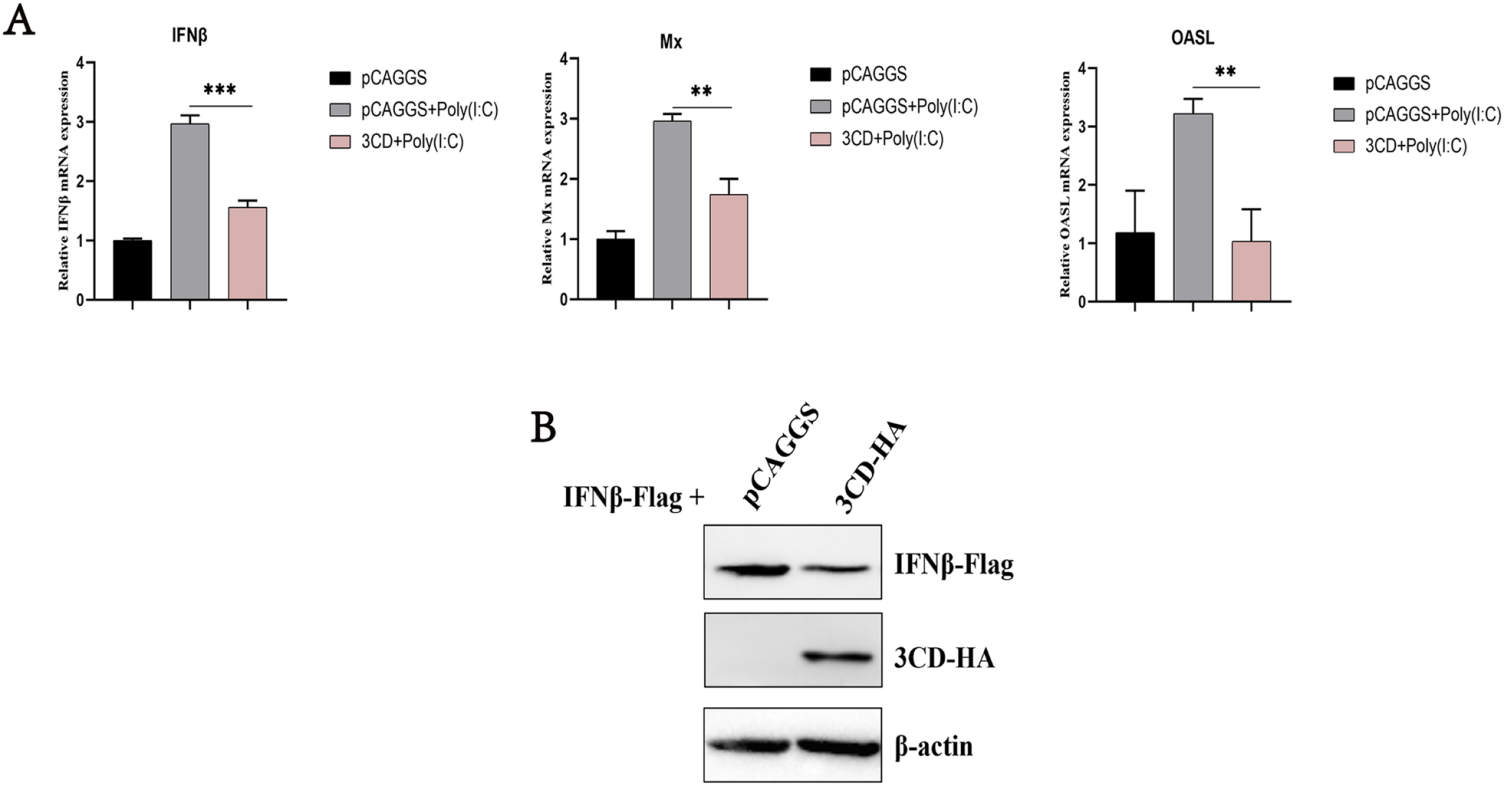
**
Effect of the 3CD protein on the IFNβ signaling pathway.A** pCAGGS-3CD-HA and Poly(I:C) were cotransfected into 24-well plate cells (transfection ratio of 1:1), and the cell samples were collected 36 h after transfection and the total RNA was extracted from the samples. Quantitative PCR analysis of the effect of the 3CD protein on poly(I:C)-induced IFN-β and IFN-β-induced MX and OASL mRNA levels. *P < 0.05, **P < 0.01, ***P < 0.001, compared with the control group. **B** 0.6 µg pCAGGS-3CD-HA and 0.4 µg pCAGGS-IFNβ-Flag were co-transfected into cells in 12-well plates, and cell samples were collected 36 h after transfection to detect the expression of IFNβ protein.

Previous studies have shown that the DHAV-1 3C protein can inhibit the IFNβ upstream signaling pathway by targeting the IRF7 protein [26]. The presence of 3CD protein as a precursor to 3C protease and 3D polymerase is unclear as to whether it also has the immune evasion function of 3C protein. To explore the specific role of the 3CD protein in the immune response, this study first explored the effect of 3CD on the Poly(I:C)- induced IFNβ pathway. The results showed that 3CD protein significantly inhibited the upregulation of mRNA levels of IFN-β and IFN-β-induced increases in Mx and OASL mRNA levels (Figure 1A) and that 3CD also had a slight effect on the protein expression of IFNβ (Figure 1B).

### The 3CD protein interacts with the IRF7 and inhibits the expression of IRF7 protein

**Figure 2:**
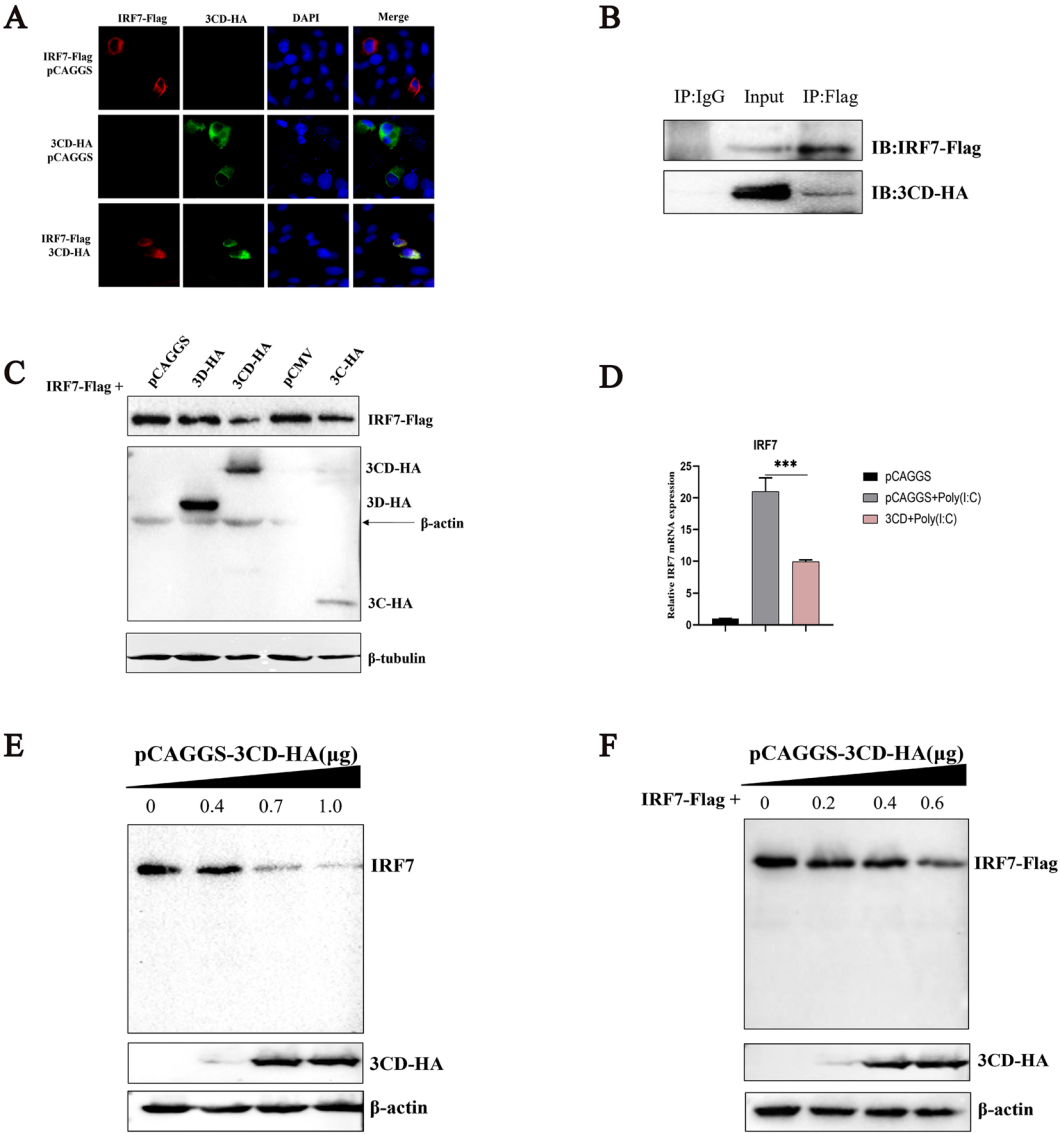
**The 3CD protein interacts with IRF7 protein and inhibits the expression of IRF7 protein.A** pCAGGS-3CD-HA and pCAGGS-IRF7-Flag were co-transfected or both were separately co-transfected with pCAGGS into 24-well plates (transfection ratio of 1:1), and cell samples were collected 36 h after transfection for indirect immunofluorescence experiments to observe the intracellular localization of the 3CD protein and IRF7 protein. **B** Cells were grown in 6-well plates and each well was transfected with a total of 1 µg pCAGGS-3CD-HA and 1 µg pCAGGS-IRF7-Flag. Cells were lysed 36 h after transfection, and lysates were immunoprecipitated with mouse anti-Flag or mouse IgG antibodies and subjected to Western blotting. **C** 0.4 µg pCAGGS-IRF7-Flag was co-transfected into 12-well plates with 0.6 µg pCAGGS-3CD-HA, pCAGGS-3D-HA, pCMV-3 C-HA or empty, respectively, and cell samples were collected 36 h after transfection to detect the expression of IRF7 protein. **D** Similar transfection and quantitative PCR experiments were performed to analyze the effect of 3CD protein on Poly(I:C)-induced IRF7 mRNA levels as described in Figure 1A. * *P* < 0.05, ** *P* < 0.01, *** *P* < 0.001, compared with the control group. **E** DEFs were grown in 12-well plates, and different doses of 3CD-HA expression plasmids were transferred into the cells (the amount transfected into each group was 1 µg, and pCAGGS was used to supplement the insufficient group). The expression of 3CD protein and endogenous IRF7 protein was detected by Western blotting at 36 h. **F** DEFs were grown in 12-well plates, and 0.4 µg of pCAGGS-IRF7-Flag was co-transfected with different doses of pCAGGS-3CD-HA (the amount transfected into each group was 1 µg, and pCAGGS was used to supplement the insufficient group). 36 h after transfection, cell samples were collected, and the protein expressions of 3CD and IRF7 were detected.

Since the 3CD protein is capable of inhibiting the IFNβ upstream signaling pathway, and the 3 C protein has been found to interact with the IRF7 protein in previous studies, it seems reasonable to expect that the 3CD protein will also bind the IRF7 protein. Here we first tested this conjecture using indirect immunofluorescence and co-immunoprecipitation experiments. The experimental results showed a clear co-localization of the 3CD protein with the IRF7 protein in the cytoplasm (Figure 2A) and a 3CD protein band was detectable by Western blotting when IRF7-Flag was used as a pull-down protein, indicating that 3CD could also interact with the IRF7 protein (Figure 2B). Then, we explored the effects of 3C, 3CD, and their precursor protein 3CD on the expression of exogenous IRF7 protein. The results showed that both 3 C and 3CD significantly inhibited the expression of IRF7 protein, whereas no significant changes were observed for 3D protein (Figure 2C). Next, we explored the effect of 3CD protein on IRF7 protein mRNA and measured the abundance of IRF7 mRNA in cells transfected with 3CD-HA under Poly(I:C) stimulation by qPCR. The results showed that the 3CD protein significantly inhibited the up-regulation of IRF7 mRNA level induced by Poly(I:C) (Figure 2D). To further determine the effect of 3CD on IRF7 protein expression, dose-response experiments were performed, and it was observed that both exogenous and endogenous IRF7 protein levels were reduced in a dose-dependent manner by the expression of 3CD protein. However, no cleavage bands were observed using the anti-IRF7 and Flag antibodies (Figures 2E and F). These results indicate that, like the 3 C protein, the 3CD protein also interacts with and inhibits IRF7 protein expression.

### The 3CD protein does not directly affect the localization and distribution of IRF7 protein

**Figure 3:**
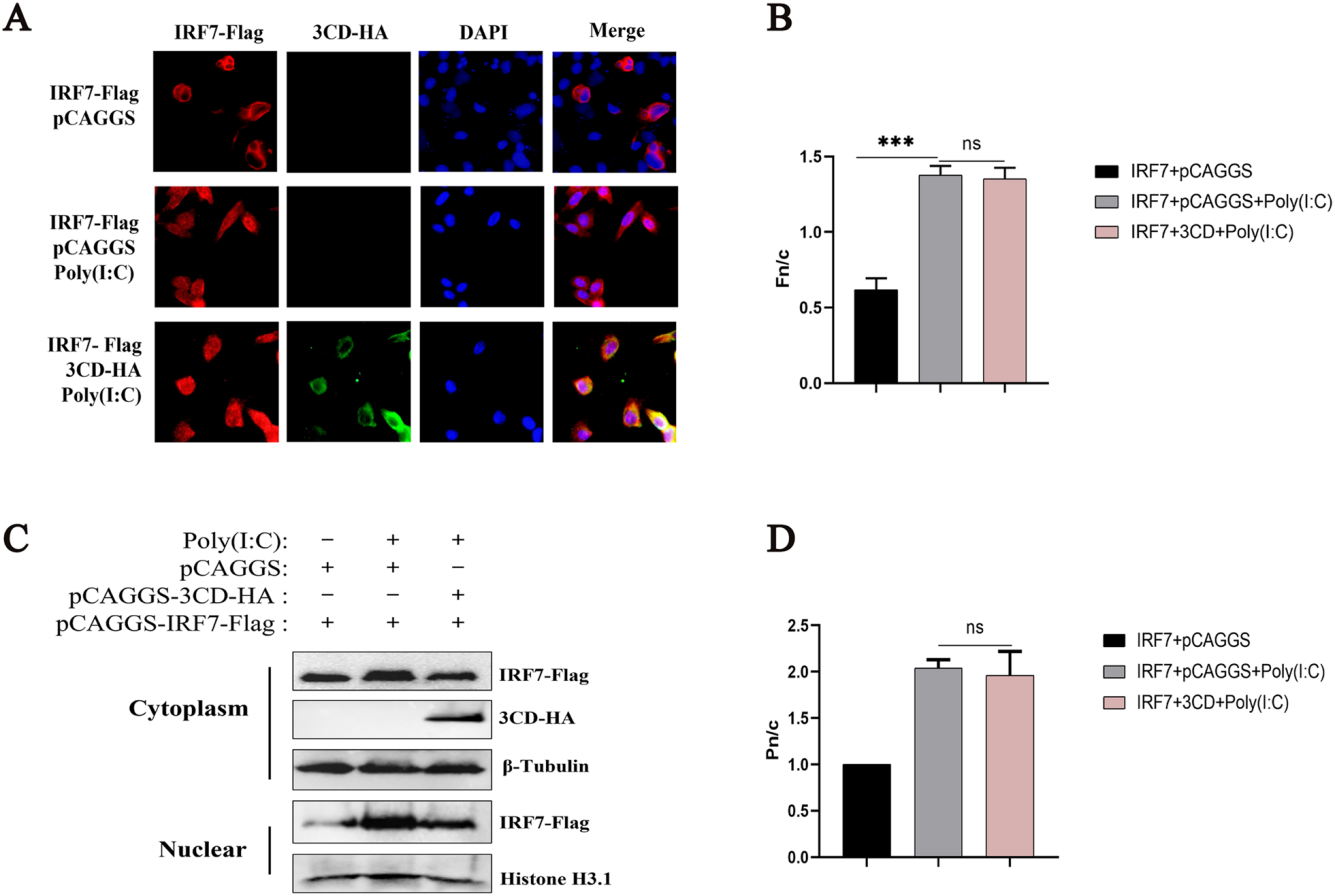
**Effect of the 3CD protein on the nuclear translocation of the IRF7 protein.A** Three experimental groups were set up, both groups I and II were co-transfected with 0.3 µg pCAGGS-IRF7-Flag and 0.5 µg pCAGGS; the third group was transfected with 0.3 µg pCAGGS-IRF7-Flag and 0.5 µg pCAGGS-3CD-HA, and transfect the plasmids into cells in a 24-well plate. 24 h after transfection groups II and III were stimulated with 5 µg/µL Poly (I:C) for 12 h, and cell samples were collected for indirect immunofluorescence experiments. **B** Images such as those in panel A were subjected to image analysis (see Materials and methods), where > 30 cells for each sample were analyzed to determine Fn/c, the fluorescence in the nucleus relative to that in the cytoplasm. Results are means ± standard errors of the mean (*n* ≥ 30). **C** DEFs were cultured in 6-well plates, grouped and transfected as described above for group A (transfection ratio of 3:2), 24 h after transfection groups 2 and 3 were stimulated with 20 µg/µL Poly(I:C) for 12 h, and cell samples were collected for Western blot analysis. **D** Gray value analysis was performed on the bands in C with ImageJ software to determine the ratio of Pn/c: Pn/c = Pn/Pc, Pn (relative protein level of IRF7 in the nucleus) = gray value of nuclear IRF7 protein/ Histone H3.1, Pc (relative protein level of IRF7 in the cytoplasm) = gray value of cytoplasmic IRF7 protein/β-tubulin. Where the data in each group were normalized relative to the control group. Values represent the mean (± SD) from three independent experiments. NS: not significant, * *P* < 0.05, ** *P* < 0.01, *** *P* < 0.001, compared with the control group.

Localized in the cytoplasm, IRF7 is activated to enter the nucleus to activate the transcription of related genes, which in turn releases cytokines, interferons, or some other signaling molecules. Here, we explored the direct effect of 3CD protein on IRF7 nuclear translocation. Cells were co-transfected with pCAGGS-IRF7-Flag and pCAGGS-3CD-HA or pCAGGS. The results are shown in Figure 3A. When IRF7 was expressed with pCAGGS, it was mainly localized in the cytoplasm; when Poly(I:C) was used as the stimulator, IRF7 was localized in the cytoplasm and nucleus; when 3CD was co-expressed with IRF7 and Poly(I:C) was used as the stimulator, IRF7 was localized in the cytoplasm and nucleus, which remained basically the same as the control (group II) (Figure 3A). We then analyzed the images to determine the nuclear translocation of IRF7 based on the ratio of nuclear to cytoplasmic fluorescence (Fn/c), which showed that the 3CD protein did not alter the nuclear translocation of IRF7 protein (Figure 3B), supporting the above observations. To further verify this phenomenon, we performed a nucleocytoplasmic separation experiment. The results showed that both nuclear and cytoplasmic expression of IRF7 protein increased when stimulated with Poly(I:C) compared with transfection of IRF7 plasmid alone, whereas when 3CD and IRF7 proteins were co-expressed, the expression levels of IRF7 protein in the nucleus and cytoplasm were significantly reduced compared with those in the Poly(I:C) group (Figure 3C). Subsequently, we performed gray value analysis of the protein bands in Figure 3C to quantify the distribution of IRF7 protein in and out of the nucleus. The results showed that the ratio of relative protein levels of IRF7 protein inside and outside the nucleus (Pn/c) remained constant even in the presence of 3CD protein (Figure 3D). These results demonstrate that the 3CD protein has no direct effect on the localization and distribution of IRF7 protein.

### The 3CD protein reduces the protein expression of RIG-I and inhibits RIG-I-mediated signal transduction

**Figure 4:**
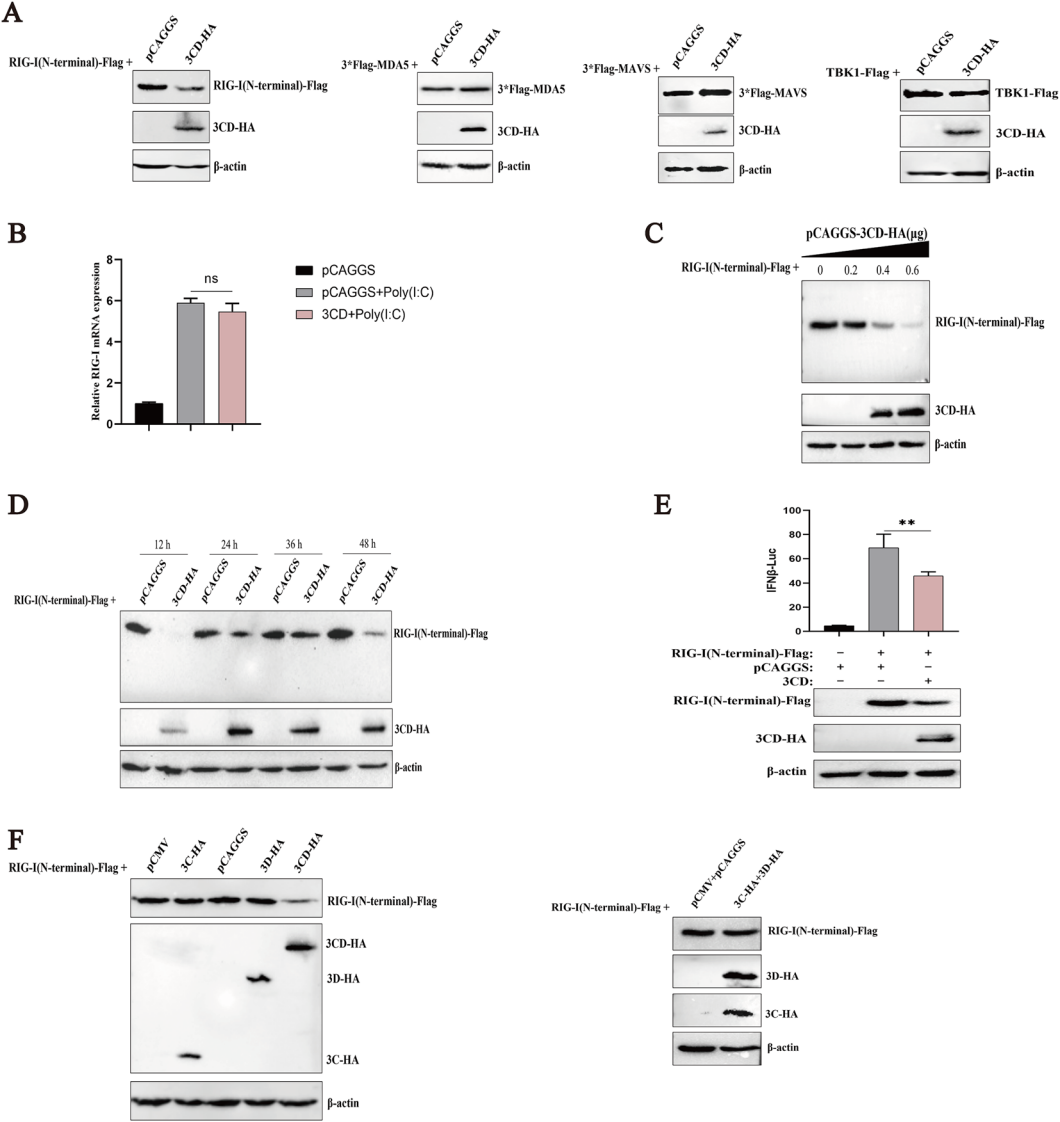
**The 3CD protein induces the reduction of RIG-I and inhibits RIG-I-mediated signal transduction.A** Cells were grown in 12-well dishes, and pCAGGS-RIG-I (N-terminal)-Flag, pCAGGS-3*Flag-MDA5, pCAGGS-3*Flag-MAVS, pCAGGS-TBK1-Flag were transfected with pCAGGS-3CD-HA or pCAGGS, respectively (transfection ratio of 2:3) and cell samples were collected for Western blot analysis at 36 h of transfection. **B** Similar transfection and quantitative PCR experiments were performed to analyze the effect of 3CD protein on Poly(I:C)-induced RIG-I mRNA levels as described in panel A of Figure 1. NS, not significant, compared with the control group. **C** DEFs were inoculated in 12-well plates and 0.4 µg of pCAGGS-RIG-I(N-terminal)-Flag was co-transfected with different doses of pCAGGS-3CD-HA (the amount transfected into each group was 1 µg, and pCAGGS was used to supplement the insufficient group). Cell samples were collected 36 h post-transfection and assayed for 3CD and RIG-I protein expression. **D** Cells in 12-well plates were co-transfected with plasmids expressing 3CD-HA (0.6 µg) or empty plasmid and RIG-I (N-terminal)-Flag (0.4 µg). Cells were collected at 12, 24, 36, and 48 h, and cell lysates were analyzed by Western blotting to detect the expression levels of RIG-I and 3CD. **E** pCAGGS-3CD-HA or pCAGGS was co-transfected with pCAGGS-RIG-I(N-terminal)-Flag, IFNβ pro-Luc, and the reference plasmid pRL-TK into 24-well plates (the transfection ratio of the first three plasmids was 2:1:1, and the amount of pRL-TK transfected was 1/20 that of IFN-β pro-Luc). 36 h after transfection, cell samples were collected. The dual-luciferase reporter system was used to detect IFN-β promoter activity. ** *P* < 0.01, ***P < 0.001, compared with control group. **F** 0.4 µg of pCAGGS-RIG-I (N-terminal)-Flag was transfected with 0.6 µg of pCAGGS-3CD-HA, pCAGGS-3D-HA, pCMV-3 C-HA or pCAGGS into cells in 12-well plates respectively, and cell samples were collected 36 h after transfection to analyze the expression of each protein.

To investigate whether 3CD protein inhibits other proteins in the upstream pathway of IFNβ, Flag-tagged MDA5, RIG-I (N-terminal), MAVS, and TBK1 expression plasmids were co-transfected with empty vector plasmid or 3CD-HA respectively, and the expression levels of each protein were examined by Western blotting. The results showed that the DHAV-1 3CD protein significantly induced a reduction in RIG-I protein expression, but not in MDA5, MAVS, and TBK1 proteins, compared with the control group (Figure 4A). To investigate whether the reduction in RIG-I was the result of a specific reduction in mRNA expression, the abundance of RIG-I mRNA in cells transfected with 3CD-HA under Poly(I:C) stimulation was measured by qPCR. The results showed no obvious change in RIG-I mRNA levels (Figure 4B). This implies that either the DHAV-1 3CD protein degraded RIG-I or that 3CD restricted RIG-I synthesis. However, the 3CD-induced reduction in RIG-I revealed a novel mechanism evolved by DHAV-1 to counteract the host antiviral response.

To further determine the effect of the 3CD protein on RIG-I protein expression, a dose-response experiment was performed. The results showed that the 3CD protein decreased RIG-I protein levels in a dose-dependent manner. However, we did not detect a cleavage band (Figure 4C). Subsequently, the kinetics of 3CD-induced reduction of RIG-I was examined. DEFs were transfected with plasmids expressing 3CD-HA and RIG-I (N-terminal)-Flag. Cells were then collected at different time points and RIG-I expression was determined by Western blotting. As shown in Figure 4D, the phenomenon of 3CD-induced reduction of RIG-I remained constant over time. The effect of 3CD on RIG-I-mediated IFNβ promoter activation was then determined by reporter gene analysis. The results showed that 3CD significantly inhibited the activation of the IFN-β promoter (Figure 4E). As 3CD is a precursor protein containing the structural domains of 3 C protease and 3D polymerase, we explored the effects of 3 C, 3D, and 3CD proteins on exogenous RIG-I protein expression. The results showed that the 3CD protein significantly inhibited the protein expression of RIG-I compared with the control, while the 3 C and 3D proteins had no significant effect (Figure 4F). We then investigated whether the combination of 3 C and 3D could affect the expression of RIG-I protein. The results showed that a simple combination of the two did not affect the expression of RIG-I (Figure 4F). These results suggest that the DHAV-1 3CD protein can inhibit the expression of RIG-I protein.

### The 3CD protein interacts with the N-terminal structural domain of RIG-I

**Figure 5:**
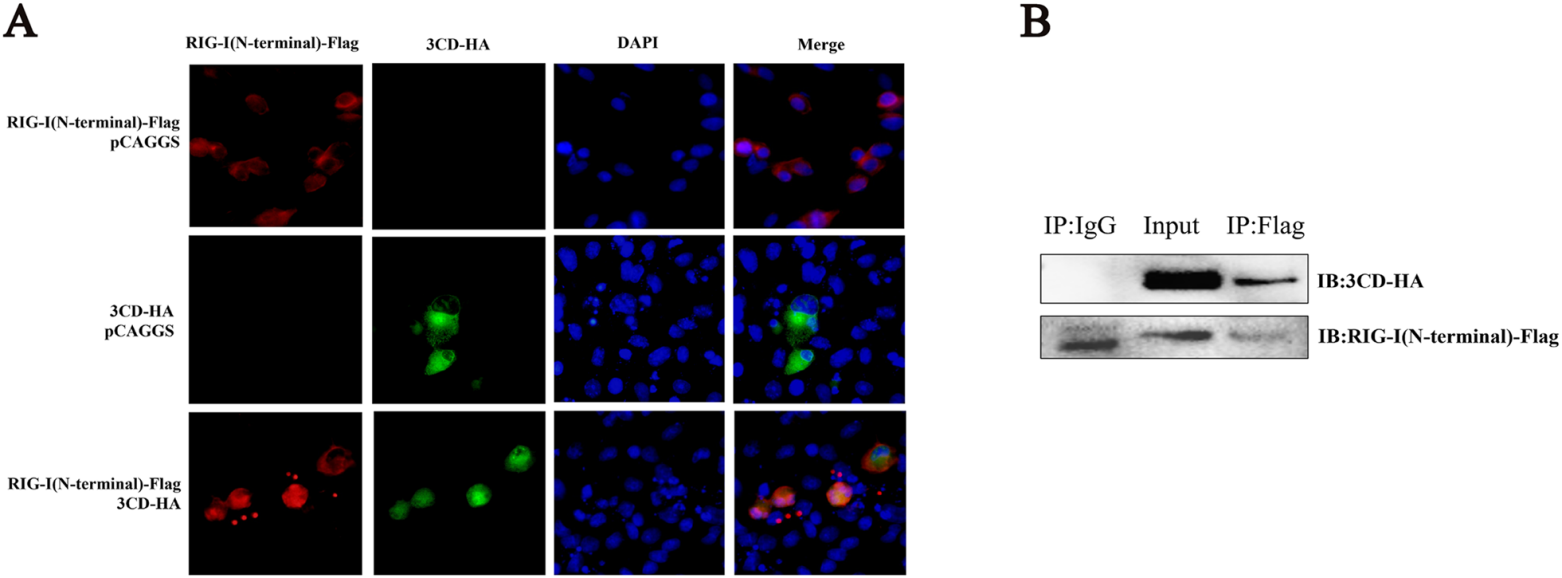
**The 3CD protein interacts with the N-terminal domain of RIG-I.A** pCAGGS-3CD-HA and pCAGGS-RIG-I (N-terminal)-Flag were co-transfected or both were co-transfected separately with empty plasmid into 24-well plates (transfection ratio of 1:1), and cell samples were collected 36 h after transfection for indirect immunofluorescence assays to observe the intracellular localization of 3CD and RIG-I. **B** Cells were seeded in 6-well plates, and 1 µg of pCAGGS-3CD-HA and 1 µg of pCAGGS-RIG-I (N-terminal)-Flag were co-transfected into monolayers. Cells were lysed 36 h after transfection, and lysates were immunoprecipitated with mouse anti-Flag or mouse IgG antibodies and subjected to Western blotting.

To investigate the possible interaction between the 3CD protein and RIG-I protein, DEFs were transfected with plasmids expressing 3CD-HA and RIG-I (N-terminal)-Flag, and indirect immunofluorescence assays and coimmunoprecipitation experiments were performed. The results showed that there was a clear co-localization of the 3CD protein with the RIG-I protein in the cytoplasm (Figure 5A) and a 3CD protein band was detected by Western blotting when pulled down with Flag antibody, indicating that the RIG-I protein could interact with the 3CD protein through the N-terminal structural domain (Figure 5B).

### The 3CD protein interferes with the interaction between RIG-I and MAVS

**Figure 6:**
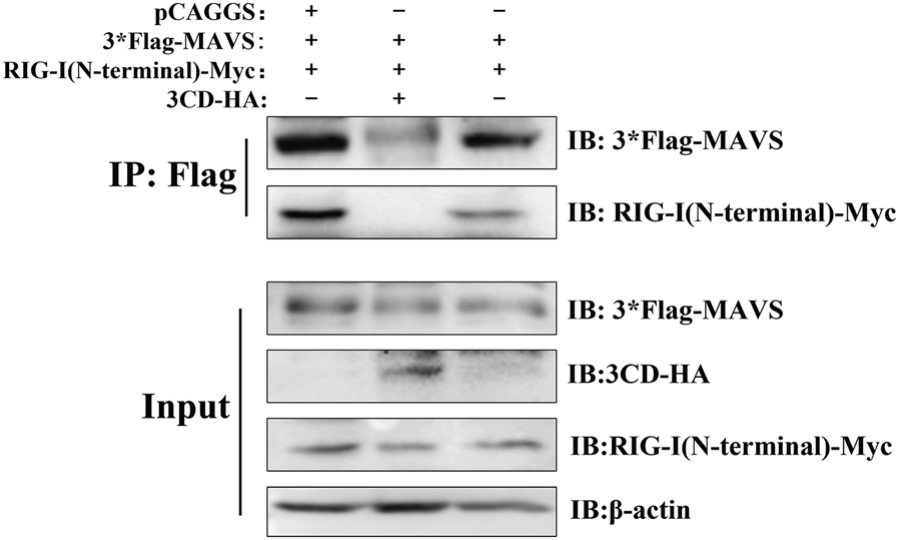
**The 3CD protein interferes with the interaction between RIG-I and MAVS.** DEFs were co-transfected with 0.8 µg pCAGGS-3CD-HA or empty plasmid, 0.6 µg pCAGGS-3*Flag-MAVS and 0.6 µg pCAGGS-RIG-I (N-terminal)-Myc, respectively, in 6-well plates. Cells were lysed 36 h after transfection and the supernatant was collected for immunoprecipitation assay using an anti-Flag antibody. RIG-I, 3CD, and MAVS were detected by Western blotting using anti-Flag, anti-Myc, and anti-HA antibodies.

MAVS acts downstream of RIG-I in signaling by binding to the activated RIG-I CARD region to form a complex, and the finding that the DHAV-1 3CD protein binds RIG-I and reduces IFN-β mRNA and protein levels induction in response to RIG-I ligand 5ʹpppRNA suggests that the 3CD protein may deregulate RIG-I-mediated signaling to downstream adapters. To investigate whether the 3CD protein abrogates the interaction between RIG-I and MAVS, we performed a co-immunoprecipitation assay. DEFs were co-transfected with 3*Flag-MAVS and RIG-I (N-terminal)-Myc as well as a 3CD-HA expression plasmid or a vector without 3CD (control). Although MAVS binding to RIG-I was readily demonstrated in the absence of the 3CD protein, no interaction was observed in the presence of the 3CD protein (Figure 6). These results suggest that the 3CD protein prevents the formation of the RIG-I/MAVS complex.

### The 3CD protein degrades RIG-I protein through the proteasome pathway

**Figure 7:**
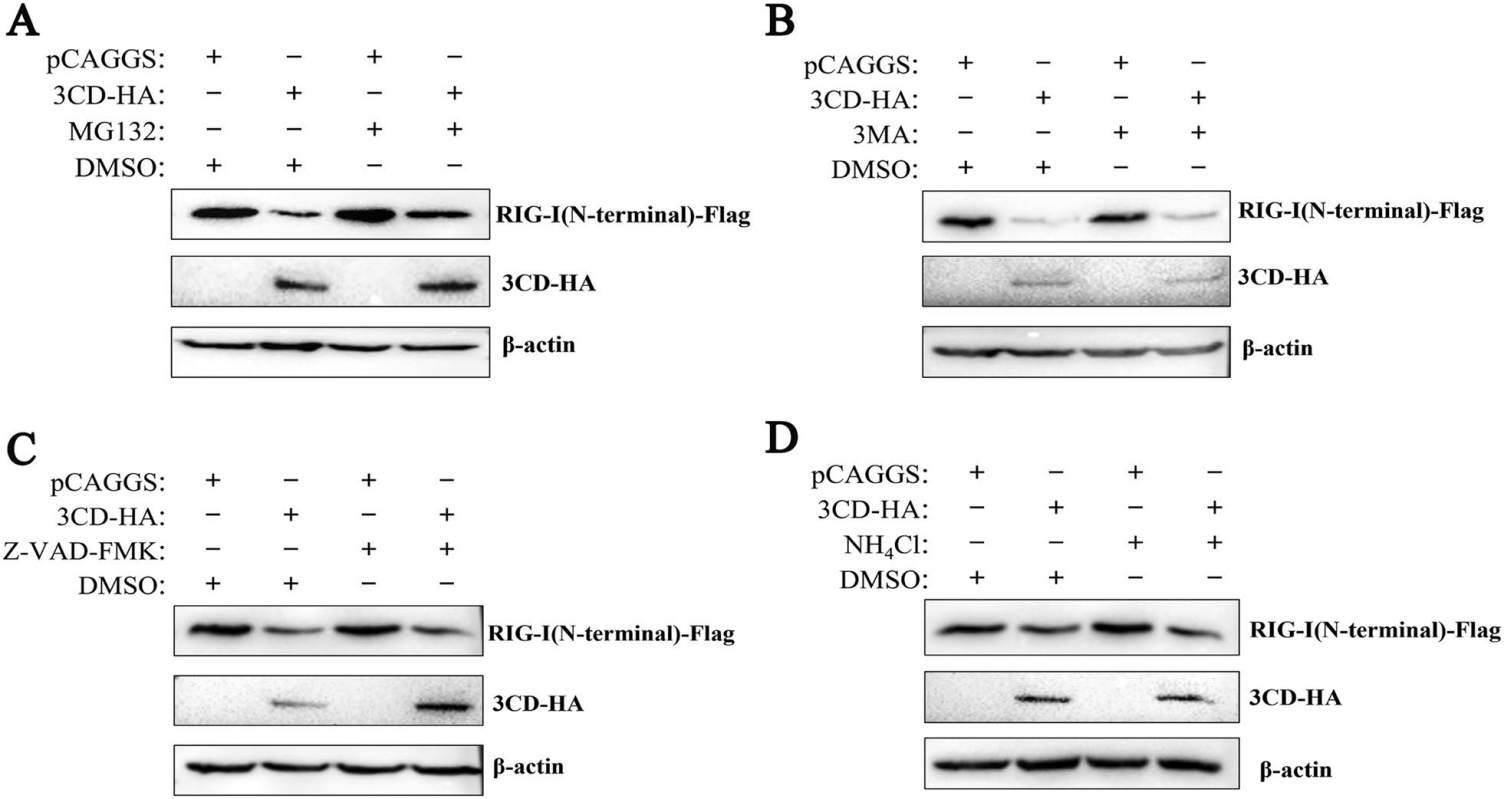
**The 3CD protein reduces RIG-I protein through the proteasome pathway.** Cells in 12-well plates were co-transfected with 3CD-HA (0.6 µg) or empty plasmid and RIG-I (N-terminal)-Flag (0.4 µg). At 24 h post-transfection, the broad-spectrum caspase inhibitor Z-VAD-FMK (50 µM), proteasome inhibitor MG132 (20 µM), autophagy inhibitor 3-MA (10 mM), and autophagy-lysosome inhibitor NH_4_Cl (10 mM) were added to the cell culture medium, while a DMSO negative control group was set up and treated for 12 h. Cell samples were collected after 12 h of treatment, and immunoblotting experiments were performed in the presence or absence of these inhibitors. The expression level of RIG-I was detected. **A** The effect of MG132 on RIG-I protein degradation. **B** The effect of 3-MA on RIG-I protein degradation. **C** The effect of Z-VAD-FMK on RIG-I protein degradation. **D** The effect of NH_4_Cl on RIG-I protein degradation.

Since the decrease in RIG-I is not the result of a specific decrease in its mRNA expression, it is speculated that 3CD may indirectly decrease the protein expression of RIG-I through the intracellular protein degradation pathway. In eukaryotic cells, protein degradation pathways are mainly divided into the autophagy-lysosomal pathway, proteasome pathway, and caspase-dependent pathway [27]. To determine whether these protein degradation pathways play a role in the DHAV-1 3CD-induced reduction of RIG-I, the proteasome inhibitor MG132, the autophagy inhibitor 3-MA, the lysosomal inhibitor NH_4_Cl, and the broad-spectrum caspase inhibitor Z-VAD-FMK were used to assess the inhibitory effect and it has been shown that these inhibitors did not affect cellular activity [26]. Cells were transfected with 3CD-HA and RIG-I(N-terminal)-Flag and maintained in the presence or absence of the inhibitors. Western blotting was performed to detect the expression of RIG-I at 36 h. The 3CD-induced reduction in RIG-I expression was not inhibited in the presence of 3-MA, NH_4_Cl, or Z-VAD-FMK (Figures 7B–D). In the presence of MG132, however, the expression of RIG-I was restored somewhat, but not completely (Figure 7A). These results suggest that the 3CD protein may partially degrade RIG-I protein through the proteasome pathway.

### The 3CD protein inhibits the RIG-I protein to promote DHAV-1 replication

**Figure 8:**
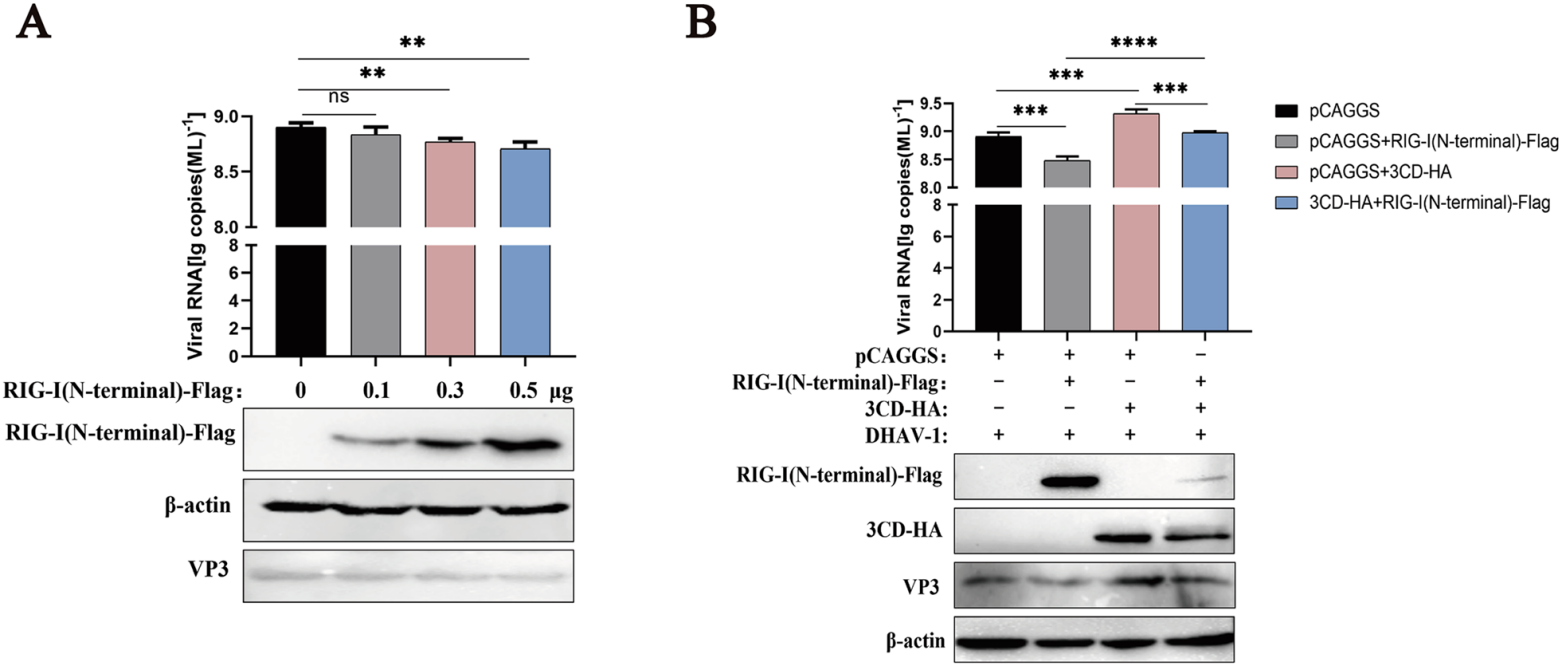
**The 3CD protein promotes DHAV-1 replication by inhibiting the RIG-I protein.A** Cells in the 24-well plate were transfected with different doses of RIG-I (N-terminal)-Flag (the amount transfected into each group was 0.5 µg, and pCAGGS was used to supplement the insufficient group), and the cells were infected with the DHAV-1 CH strain with MOI = 0.5 for 12 h after transfection. Cell samples were collected 36 h after transfection to detect the viral copy number, and the expression of VP3 and RIG-I was detected by Western blotting. **B** 0.5 µg RIG-I (N-terminal)-Flag was co-transfected with 0.5 µg 3CD-HA or co-transfected with empty plasmid respectively in 12-well plates, and cells were infected with DHAV-1 CH strain with MOI = 0.5 at 12 h. Cell samples were collected at 36 h after transfection to detect the viral copy number, and the expression of VP3, RIG-I, and 3CD was detected by Western blotting. * *P* < 0.05, ** *P* < 0.01, *** *P* < 0.001, **** *P* < 0.0001, compared with the control group.

The apparent reduction of RIG-I protein suggests a potential role for RIG-I during DHAV-1 infection. To determine whether RIG-I is involved in DHAV-1 replication, RIG-I (N-terminal)-Flag was overexpressed at different doses in this study, and cells were then infected with an equivalent amount of DHAV-1 for 24 h at 12 h to assess the effect of RIG-I on DHAV-1 replication. As shown in Figure 8A, overexpression of RIG-I significantly reduced the viral copy number and VP3 protein expression levels, and DHAV-1 replication was inhibited in a dose-dependent manner with increasing RIG-I expression. Next, we explored whether 3CD could promote viral replication by inhibiting RIG-I. 3CD-HA was cotransfected with RIG-I (N-terminal)-Flag or both separately with pCAGGS into DEFs and the cells were infected with DHAV-1. The results showed that the viral copy number and VP3 protein expression in the 3CD-HA and RIG-I (N-terminal)-Flag co-transfection group were down-regulated compared with the 3CD-HA and empty vector co-transfection group. However, compared with the co-transfection group of RIG-I (N-terminal)-Flag and empty vector, the viral copy number was significantly up-regulated, and the expression of viral protein VP3 was restored, while the expression of RIG-I protein was suppressed (Figure 8B), suggesting that the 3CD protein promotes viral replication by suppressing RIG-I expression. These results conclusively demonstrate the important antiviral role of RIG-I in DHAV-1 infection.

## Discussion

The innate immune response is the first line of defense of the host cell against viral infection. At this stage, the interferon signaling pathway plays a crucial role in enabling the expression of a set of antiviral genes responsible for antiviral effects. RLRs can recognize the invasion of a variety of viruses, including picornaviruses, so many members of the *Picornaviridae* family can suppress the immune response in host cells by inhibiting MDA5 or RIG-I. For example, coxsackievirus A16 (CV-A16), coxsackievirus A6 (CV-A6), and enterovirus D68 (EV-D68) can inhibit their interaction with MAVS by binding to MDA5 [28]. Poliovirus (PV) can disrupt the MDA5-mediated recognition system by cleaving MDA5 to establish its infection [29], which was subsequently found to be directly cleaved by the 2 A protein [15]. The 2 A proteins of CVB3, EV71, and PV were also found to have the same function in blocking the upstream signaling pathway of type I interferon by cleaving MDA5 [15]. RIG-I is cleaved by the viral protease 3 C in cells infected with PV and EMCV [30, 31]. FMDV 2B negatively regulates RLR-mediated IFN-β induction by targeting RIG-I and MDA5 [32]. This implies an important role for MDA5 and RIG-I during picornavirus infection. In addition, EV71 can block the host cell immune response by cleaving the IRF7 protein and the MAVS protein, ultimately promoting its replication and proliferation [33, 34].

**Figure 9:**
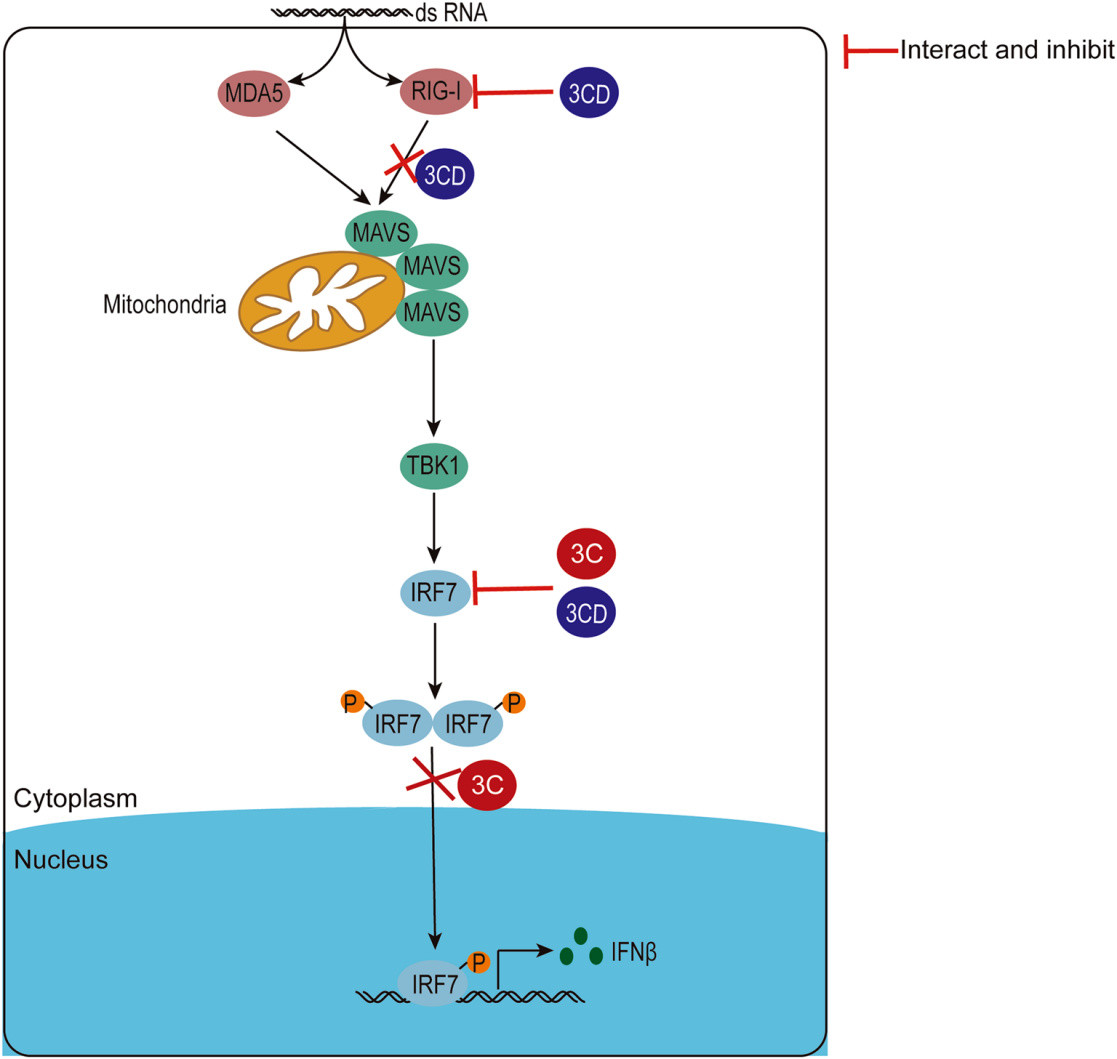
**A model of DHAV-1-mediated inhibition of IFNβ signaling pathway.** After DHAV-1 infection, both viral proteins 3 C and 3CD can inhibit the production of IFNβ by interacting with IRF7 protein and inhibiting the expression of IRF7 protein, and the 3 C protein also inhibits the nuclear translocation of the IRF7 protein, blocking interferon signaling. In addition, the 3CD protein can interact with RIG-I to inhibit its expression, and can also interfere with the formation of a complex between RIG-I and MAVS to ultimately inhibit the RIG-I-mediated signaling pathway.

DHAV-1 is a member of the *Picornaviridae* family and also can evade the type I IFN signaling pathway. The DHAV-1 3 C protein inhibits nuclear translocation of the IRF7 protein and interacts with the IRF7 protein through its N-terminus, and degrades the IRF7 protein through the caspase 3-dependent protein degradation pathway, thereby inhibiting IFNβ-mediated antiviral innate immunity to promote the replication of DHAV-1 [26] (Figure 9). In this study, we demonstrate for the first time the antagonistic role of the DHAV-1 3CD protein in the host innate immune response. Our study found that overexpression of 3CD protein significantly reduced the mRNA levels of IFNβ, Mx, and OASL, and also had an inhibitory effect on the protein expression of IFNβ. This inhibition may result from the 3CD protein directly targeting IFNβ itself, or it may be that 3CD inhibits the production of IFNβ by acting on target molecules upstream of the IFNβ signaling pathway (Figure 1). Since 3CD is a precursor to the 3 C protease and the 3D RNA-dependent RNA polymerase, can function as a protease, and is known to cleave certain cleavage sites more efficiently than 3 C in PV and FMDV [35–37], it was speculated that 3CD may have some of the functions of 3 C. Our experimental results showed that 3CD could also interact with the IRF7 protein and that both 3CD and 3 C proteins significantly inhibited the expression of IRF7 compared with the 3D protein. Furthermore, 3CD was able to inhibit the Poly(I:C)-induced upregulation of IRF7 mRNA level as well as inhibit IRF7 protein expression in a dose-dependent manner (Figure 2), consistent with the changes induced by expression of 3 C proteins in previous studies. This suggests that 3CD has the partial function of 3 C protease, both of which can interact with and inhibit the expression of IRF7 protein. However, we could not detect a cleavage band during the reduction of IRF7 protein expression caused by 3CD protein, so whether 3CD antagonizes IRF7 is related to its protease activity needs more experimental verification.

The nuclear translocation of IRF7 after activation is critical for its transcriptional activation function, and since the DHAV-1 3 C protein has been shown to inhibit the entry of IRF7 protein into the nucleus, we proceeded to study the effect of the 3CD protein on the localization of the IRF7 protein. We found that the presence of 3CD protein did not alter the localization of IRF7 protein in the nucleus and cytoplasm (Figures 3A and B). Although the inhibition of IRF7 protein expression by 3CD protein reduced the expression of IRF7 protein in both the cytoplasm and the nucleus, the proportion of IRF7 distribution inside and outside the nucleus remained unchanged (Figures 3C and D), indicating that the 3CD protein did not directly affect the nuclear entry of IRF7, unlike the phenomenon seen with 3 C protein. IRF7 can be phosphorylated and activated through two distinct pathways. TLR3 and cytoplasmic PRRs, such as sensors of poly(dA-dT), act through TBK1 and IKKε, while the TLR8 and TLR9 activate IRF7 using a signaling pathway independent of TBK1 and IKKε, but involving MyD88 and IKKα [38–40]. Therefore, it is speculated that 3 C may affect the nuclear entry of IRF7 by affecting other proteins of the above two pathways or by affecting the phosphorylation of IRF7, a function that 3CD does not have. Another possible reason is the difference in the cellular localization of the 3 C and 3CD proteins. In a study on rhinovirus (HRV), it was found that the 3 C protein has a smaller molecular weight, compared to 3CD, which has free access to the nucleus and is therefore present in both the nucleus and cytoplasm, whereas the 3CD protein is a large molecule that is more restricted in its free access to the nucleus without external forces and is mainly present in the cytoplasm [25]. However, even if the second conjecture is correct, the link between the localization of 3 C and 3CD and the localization of IRF7 needs further investigation. Thus, although the 3 C and 3CD proteins behave differently in influencing IRF7 entry into the nucleus, it appears that 3CD, as a precursor protein of 3 C, can perform some of the functions of 3 C proteins in terms of both interacting with IRF7 and inhibiting its expression.

RIG-I and MDA5 are representative receptors in the cytoplasm used to detect viral RNA and trigger antiviral responses leading to type I interferon production, as well as being key receptors for sensing picornaviruses viral RNA, but the interactions between RIG-I, MDA5, and DHAV-1 are currently relatively unknown. Here, we demonstrate that the DHAV-1 3CD protein significantly inhibits the expression of RIG-I protein, while having no apparent effect on the remaining proteins. And studies showed that 3CD inhibited the expression of RIG-I in a dose-dependent manner, and this inhibition remained unchanged for a long time. In addition, we found that only the 3CD protein inhibited the expression of RIG-I protein, but not the individual protein 3 C or 3D or a combination of the two after cleavage (Figure 4). It is conjectured that the inhibitory effect of 3CD requires both the protease and polymerase structural domains, and also needs to form a certain conformation, and the simple combination of the 3 C and 3D cannot form the conformation required for the 3CD precursor to target RIG-I, nor can it allow for conformational changes. A similar phenomenon has been reported in HAV infection. Qu et al. found that TIR domain-containing adaptor inducing interferon-β (TRIF) is proteolytically cleaved by the 3CD protein, but not by the mature 3 C protease or the 3ABC precursor that degrades MAVS. 3CD-mediated degradation of TRIF depends on the cysteine ​​protease activity of the 3 C and downstream 3D^pol^ sequences, rather than the 3D polymerase activity. The results of mutational studies suggest that the 3D^pol^ sequence modulates the substrate specificity of the upstream 3 C protease when fused to it in cis in 3CD, allowing 3CD to target cleavage sites not normally recognized by 3C^pro^ [41]. However, whether the DHAV-1 3CD protein inhibits the expression of RIG-I protein depends on its protease activity to directly cleave RIG-I or affect RIG-I through other indirect pathways, or both pathways are not yet determined. Therefore, whether the DHAV-1 3CD protein targets RIG-I in a similar manner to that reported above requires further study and cannot be concluded at this time.

Through indirect immunofluorescence and co-immunoprecipitation experiments, we confirmed that there is an interaction between 3CD and RIG-I protein and that RIG-I can interact with 3CD through its N-terminal structural domain (Figure 5). This is the first time that an interaction between the picornavirus 3CD protein and the RIG-I protein has been found. In previous literature reports, EV71 3 C was found to bind to RIG-I through the caspase recruitment domain [17], and this interaction interfered with the formation of a functional complex of RIG-I with MAVS and TBK1. Our study also showed that the interaction of 3CD with RIG-I interfered with the formation of the RIG-I-MAVS-shaped complex (Figure 6). Since the 3CD protein does not affect the RIG-I protein expression dependent on its down-regulation at the transcriptional level, it is speculated that the 3CD protein may indirectly reduce the amount of RIG-I protein by activating the protein degradation pathway in cells. In eukaryotic cells, the main protein degradation pathways are the lysosomal pathway, proteasome pathway, and caspase-dependent pathway [27]. Several examples of target protein degradation via these pathways have been reported for picornaviruses. EV71 can cleave MDA5 in a caspase-dependent manner [42, 43], and it has subsequently been reported that EV71 can degrade the nuclear localization signaling receptor KPNA1 in a caspase 3-dependent manner [27], and its 2A protein can also degrade the interferon receptor IFNAR1 in a caspase 3-dependent manner [44]. PV can degrade MDA5 in a proteasome and caspase-dependent manner [45]. Through experiments, we have identified for the first time that the DHAV-1 3CD protein in picornaviruses may partially degrade the RIG-I protein through a proteasome-dependent pathway (Figure 7), but this has not ruled out the possibility of direct cleavage of RIG-I by 3CD, and a more detailed mechanism remains to be investigated in more depth.

In this study, we demonstrate for the first time the antiviral activity of RIG-I against DHAV-1. We demonstrated that the RIG-I protein inhibited DHAV-1 replication in a dose-dependent manner, while 3CD effectively promoted viral replication by inhibiting RIG-I (Figure 8). Our experimental results identify the antiviral effect of RIG-I during DHAV-1 infection and describe for the first time a novel mechanism by which the DHAV-1 3CD protein induces a reduction in RIG-I and counteracts the antiviral effects induced by RIG-I.

In summary, our findings suggest that the DHAV-1 3CD protein can target IRF7 and RIG-I proteins to promote viral replication (Figure 9). Like the 3 C protein, 3CD can also interact with IRF7 and inhibit the expression of IRF7, however, 3CD does not directly affect the entry of IRF7 into the nucleus. Furthermore, 3CD can interact with RIG-I protein, interfere with the formation of the complex between RIG-I and MAVS, and inhibit the protein expression of RIG-I through the proteasome pathway, thereby inhibiting RIG-I-mediated signaling. This is the first report of the interaction between the picornavirus 3CD protein and IRF7 protein, and the 3CD protein and RIG-I protein, revealing a novel mechanism for evading the innate immune response to promote the self-replication during DHAV-1 infection. These results elucidate part of the pathogenesis of DHAV-1 and provide fundamental insights for the development of novel vaccines or therapeutics.
